# Supplementary material for: Identification of photoperiod-induced specific miRNAs in the adrenal glands of Sunite sheep (Ovis aries)
Source: Front Vet Sci. 2022 Jul 22;9:888207. doi: 10.3389/fvets.2022.888207 (PMC9354845; doi:10.3389/fvets.2022.888207)
Supplement: Supplementary file 1 [file Data_Sheet_1.ZIP › Supplementary file/miRDeep2 file.zip!/miRDeep2 file/Novel_Structure/SPLP42A3/Novel_411.pdf]

Provisional ID	: NC_019464.2_16037
Score total	: 0.7
Score for star read(s)	: -1.3
Score for read counts	: 0
Score for mfe	: 0.4
Score for randfold	: 1.6
Score for cons. seed	:
Total read count	: 45
Mature read count	: 45
Loop read count	: 0
Star read count	: 0

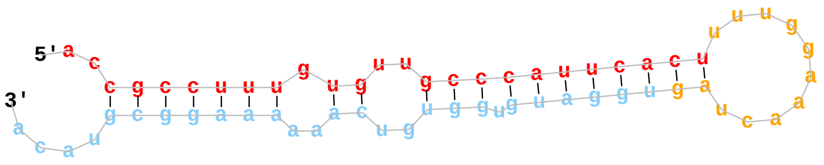

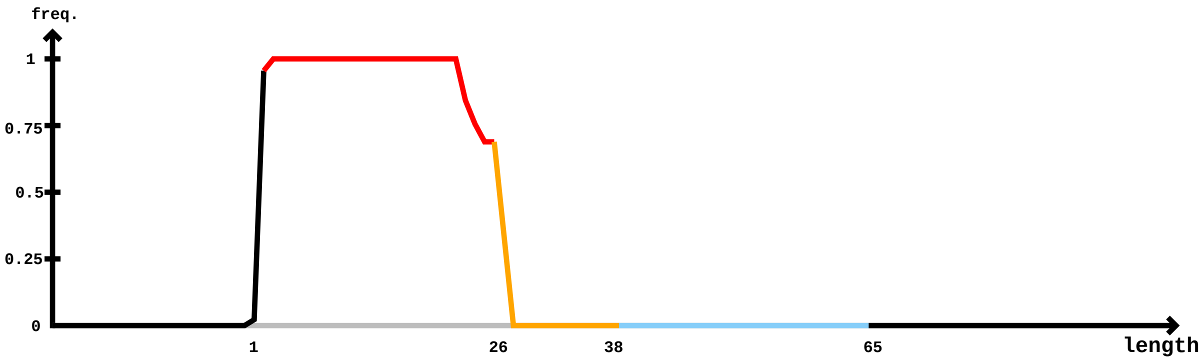

Mature **Star**

```
5'- auuccuuaugcgguuuucuuaccgccuuuguguugcccauucacuuuuggaaacuaguggaugugguguccaaaaaaggcguacacaaaacacuuucgcagcuuuuuuuccugccc -3'    exp
    ............(((((.....(((((((.((..(((((((((((..........)))))))).)))..))..))))))).....)))))......((((.........))))..   reads   mm   sample
    ...................uaccgccuuuguguugcccauuc.........................................................................   1       0    seq
    ....................accgccuuuguguugcccauu..........................................................................   7       0    seq
    ....................accgccuuuguguugcccauuc.........................................................................   3       0    seq
    ....................accgccuuuguguugcccauuca........................................................................   3       0    seq
    ....................accgccuuuguguugcccauucacu......................................................................   21      0    seq
    ....................accgccuuuguguugcccauucacuu.....................................................................   8       0    seq
    .....................ccgccuuuguguugcccauucacu......................................................................   1       0    seq
    .....................ccgccuuuguguugcccauucacuu.....................................................................   1       0    seq
```
